# Supplementary material for: Family-wide analysis of human macrodomains reveals novel activities and identifies PARG as most efficient ADPr-RNA hydrolase
Source: Commun Biol. 2025 Mar 18;8:453. doi: 10.1038/s42003-025-07901-7 (PMC11920425; doi:10.1038/s42003-025-07901-7)
Supplement: Supplementary file 1 — Supplementary Information [file 42003_2025_7901_MOESM1_ESM.pdf]

Supplementary Figure 1

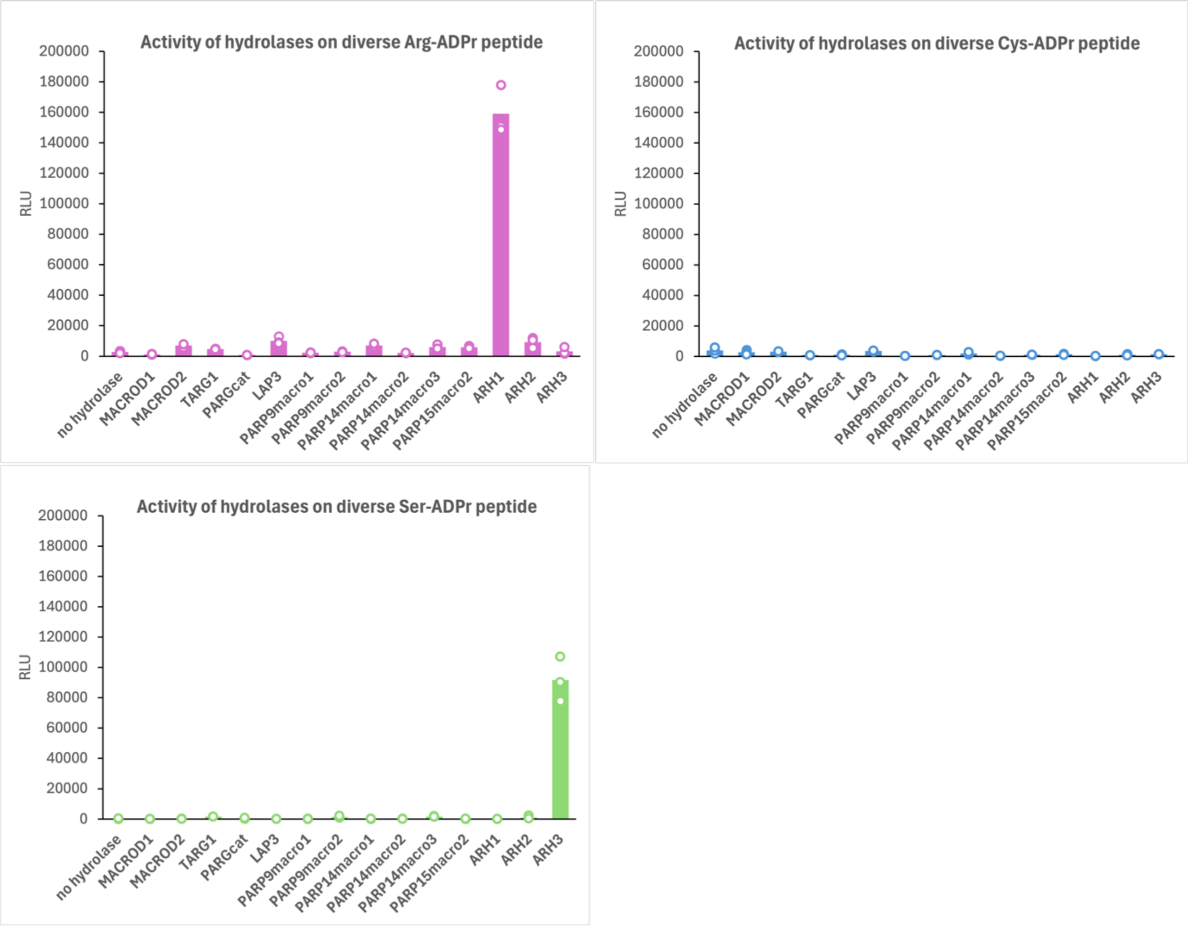

Supplementary Figure 1. AMP-Glo assay on serine-, arginine- and cysteine-ADPr peptides. An AMP-Glo assay was performed using specific ADPr-peptides, which were incubated with 1μM of the indicated hydrolases and NUDT5.
