# Supplementary material for: Family-wide analysis of human macrodomains reveals novel activities and identifies PARG as most efficient ADPr-RNA hydrolase
Source: Commun Biol. 2025 Mar 18;8:453. doi: 10.1038/s42003-025-07901-7 (PMC11920425; doi:10.1038/s42003-025-07901-7)
Supplement: Supplementary file 2 — Description of Additional Supplementary Files [file 42003_2025_7901_MOESM2_ESM.docx]

Description of Additional Supplementary Files

**File name:** Supplementary Data 1

**Description:** The uncropped gels and blots

**File name:** Supplementary Data 2

**Description:** The source data behind the graphs in the paper.
