# Supplementary material for: Family-wide analysis of human macrodomains reveals novel activities and identifies PARG as most efficient ADPr-RNA hydrolase
Source: Commun Biol. 2025 Mar 18;8:453. doi: 10.1038/s42003-025-07901-7 (PMC11920425; doi:10.1038/s42003-025-07901-7)
Supplement: Supplementary file 3 — Supplementary data 1 [file 42003_2025_7901_MOESM3_ESM.pdf]

Uncropped images belonging to Figure 2

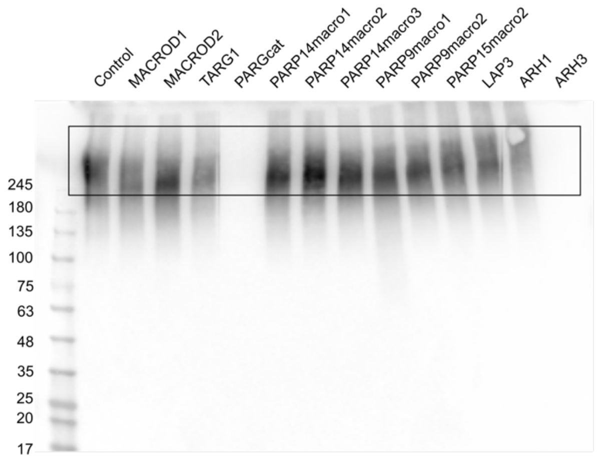

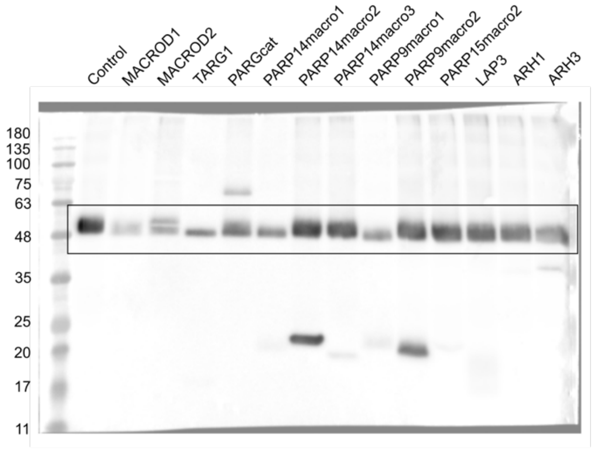

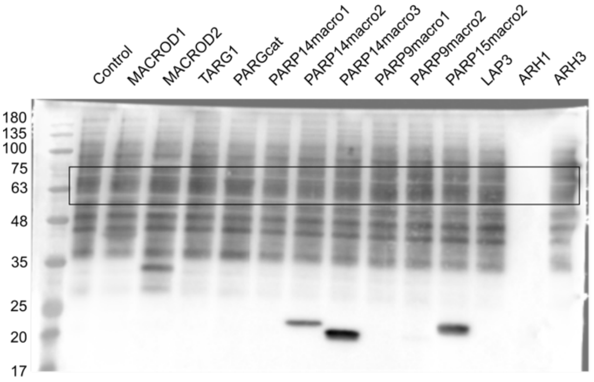

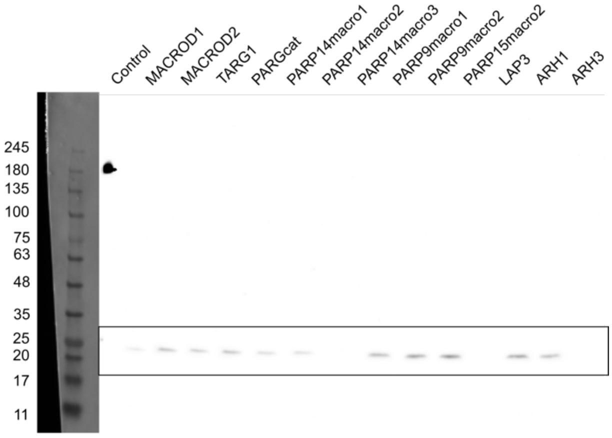

Uncropped images belonging to Figure 3

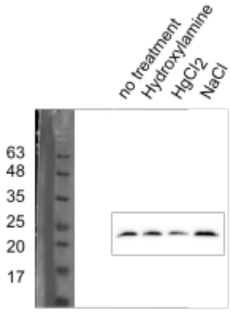

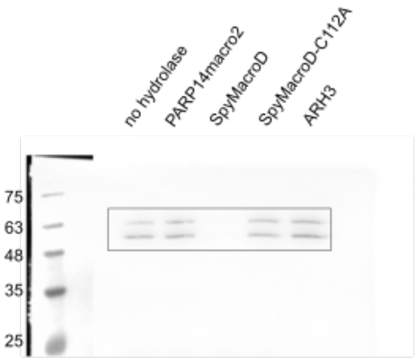

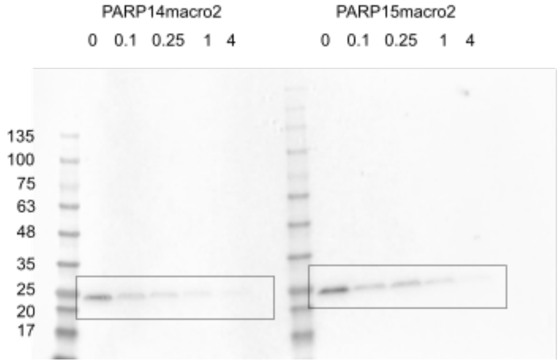

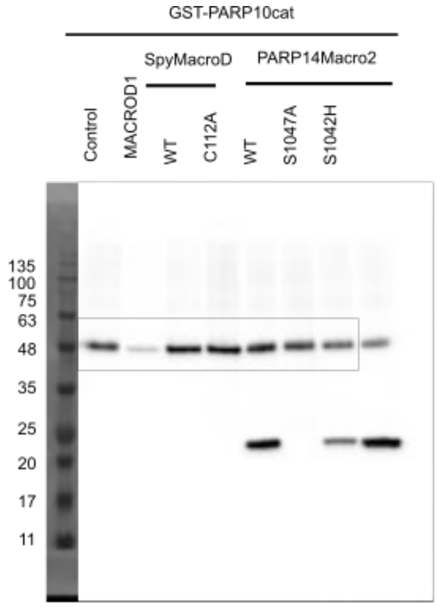

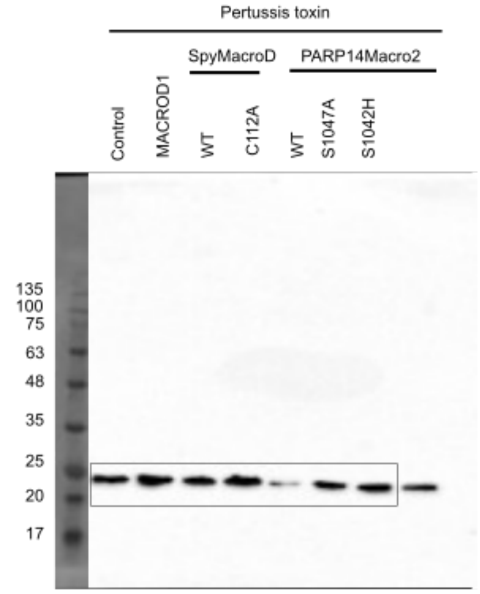

Uncropped images belonging to Figure 4

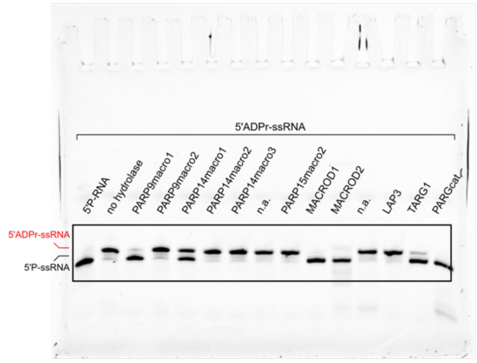

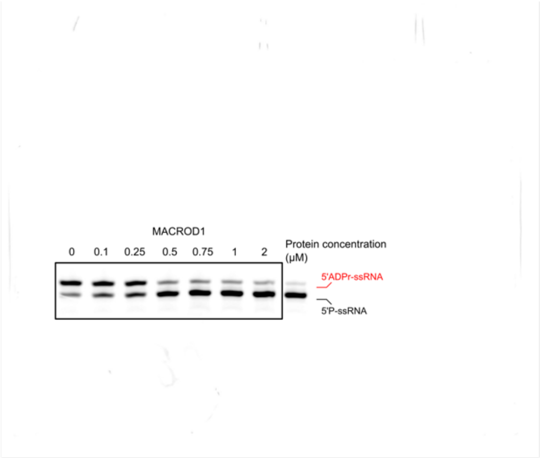

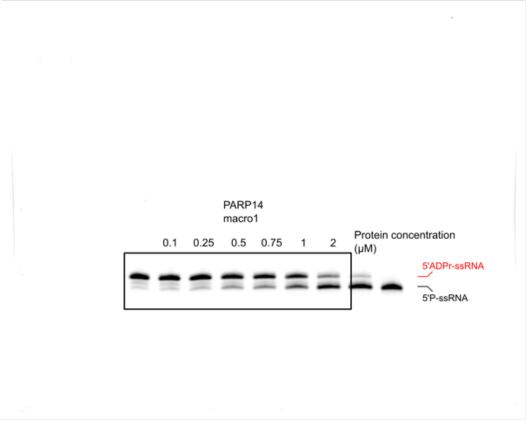

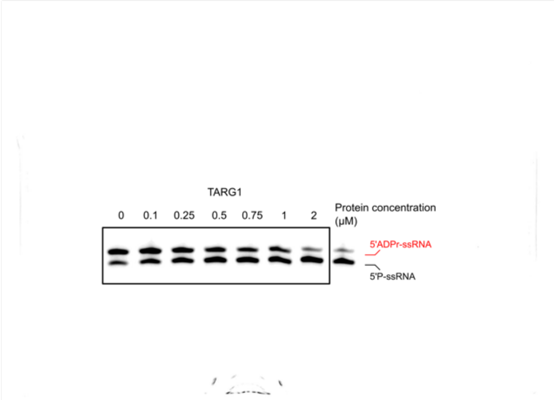

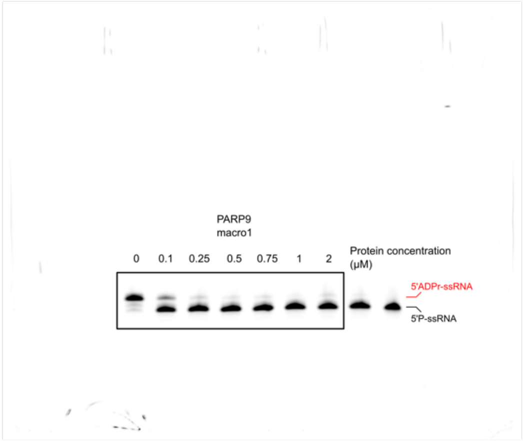

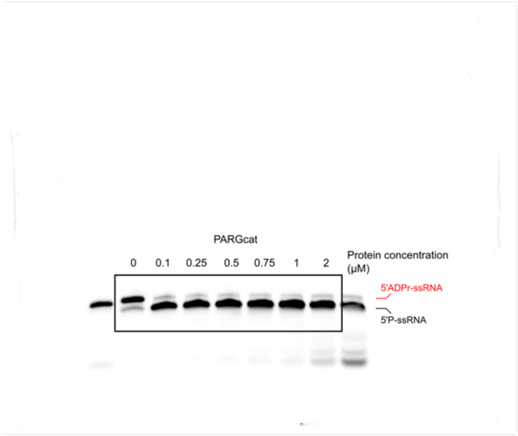

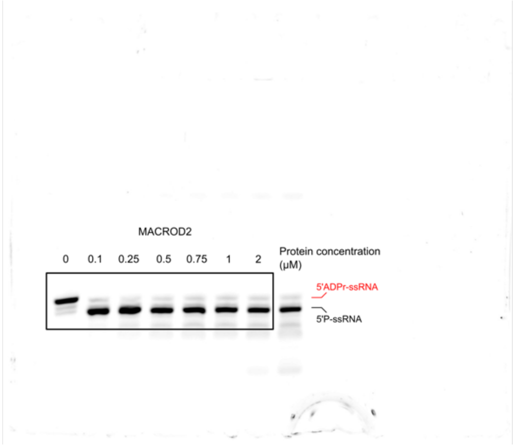

Uncropped images belonging to Figure 5

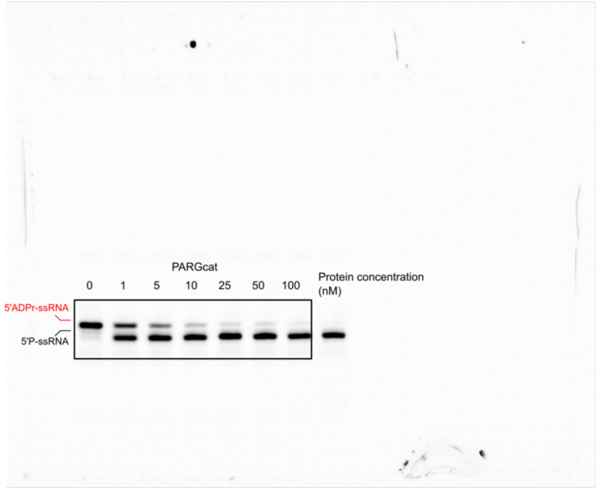
PARGcat
0 1 5 10 25 50 100
Protein concentration (nM)
5'ADPr-ssRNA
5'P-ssRNA

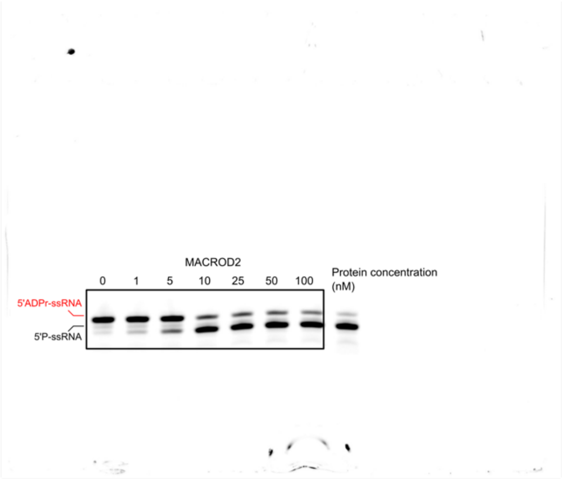
MACROD2
0 1 5 10 25 50 100
Protein concentration (nM)
5'ADPr-ssRNA
5'P-ssRNA

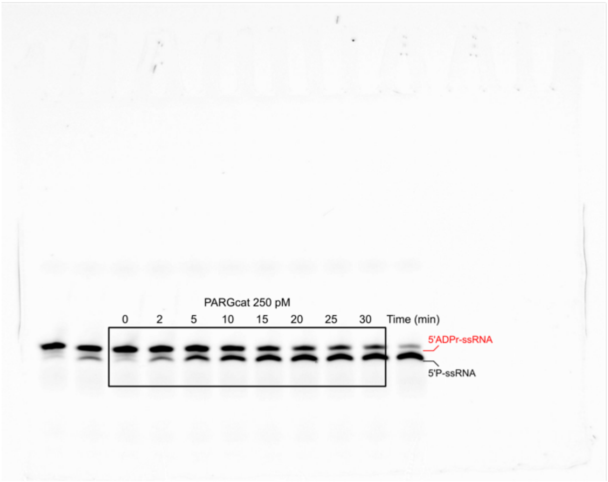
PARGcat 250 pM
0 2 5 10 15 20 25 30
Time (min)
5'ADPr-ssRNA
5'P-ssRNA

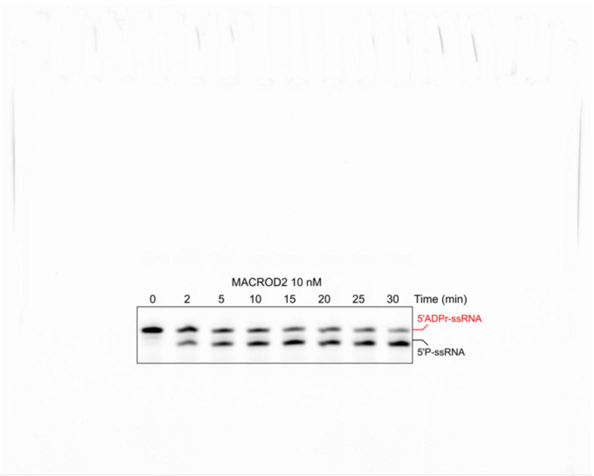
MACROD2 10 nM
0 2 5 10 15 20 25 30
Time (min)
5'ADPr-ssRNA
5'P-ssRNA

Uncropped images belonging to Figure 6

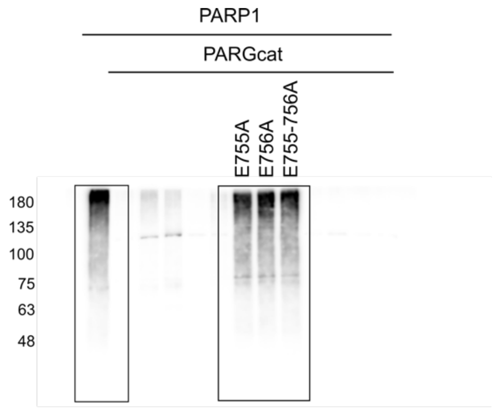

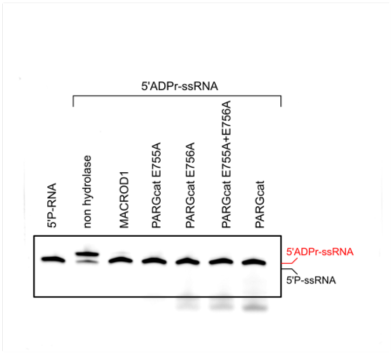

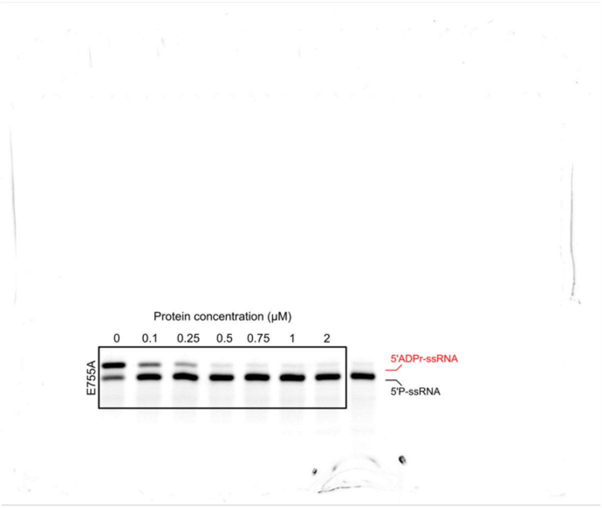

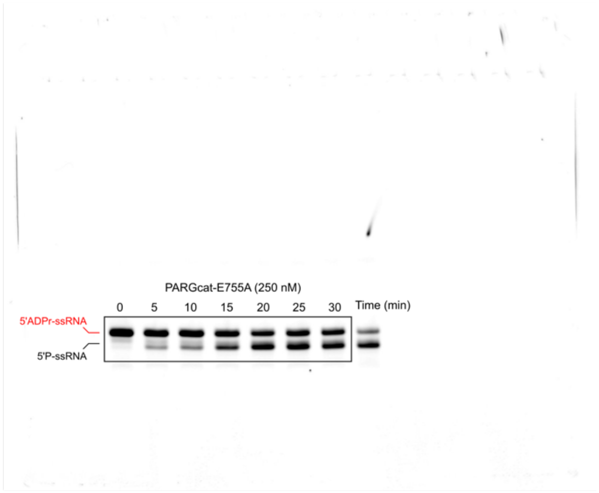

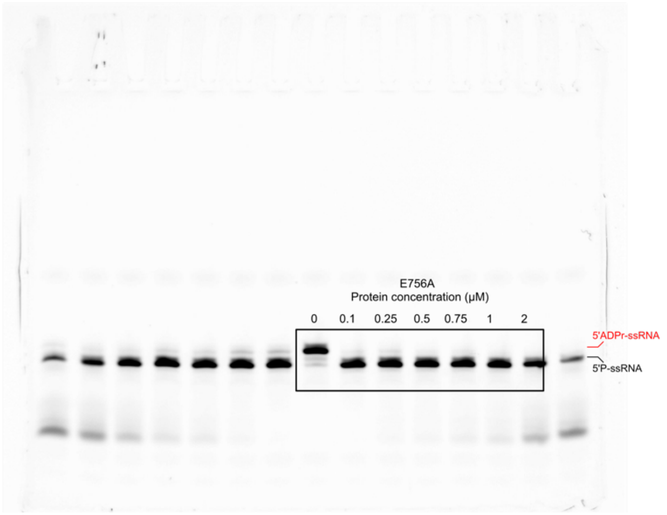

Uncropped images belonging to Figure 7

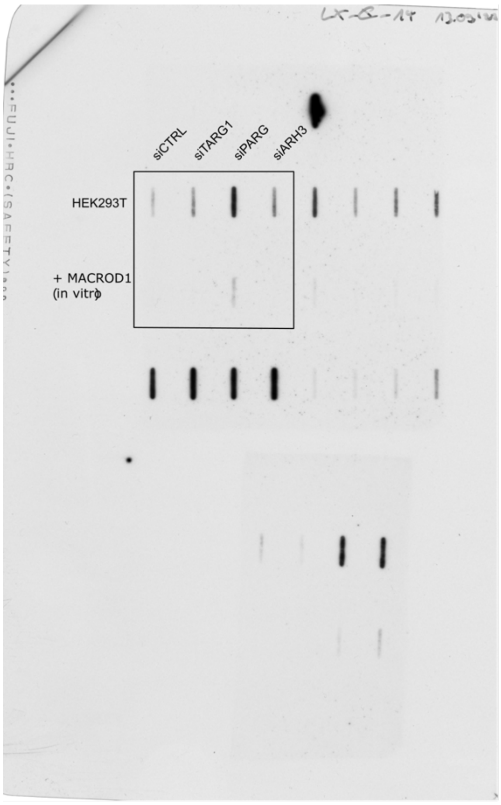
siCTRL
siTARG1
siPARG
siARH3
HEK293T
+ MACROD1
(in vitro)

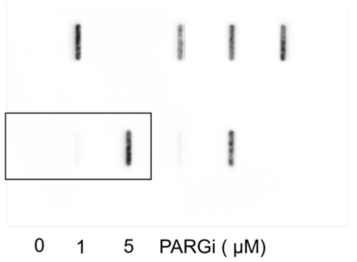
0
1
5
PARGi (μM)

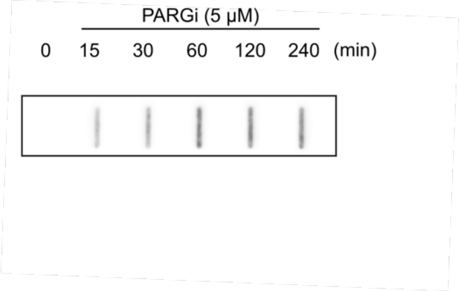
PARGi (5 μM)
0
15
30
60
120
240
(min)

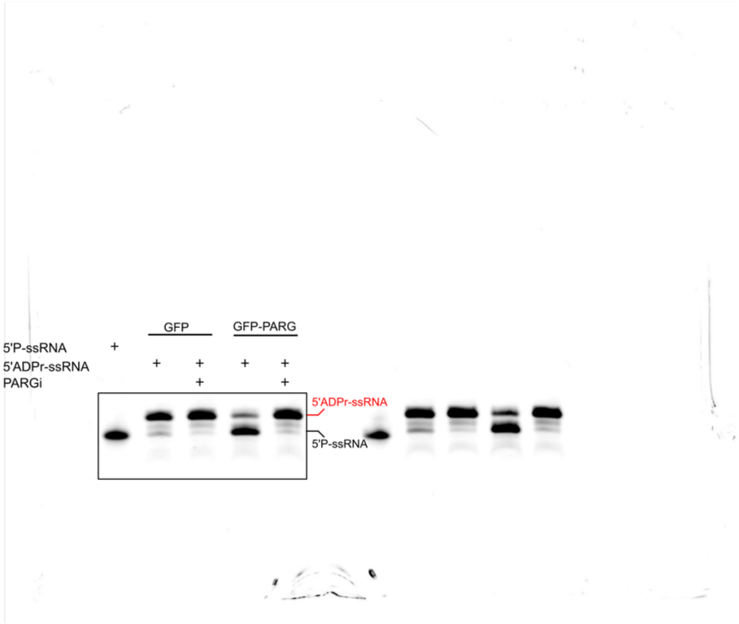
GFP
GFP-PARG
5'P-ssRNA
5'ADPr-ssRNA
PARGi
5'ADPr-ssRNA
5'P-ssRNA

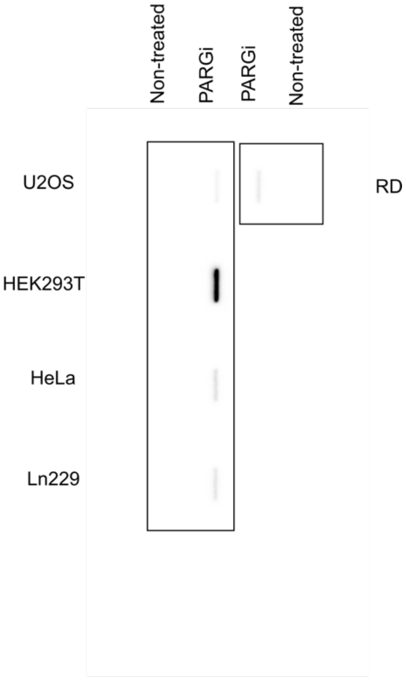
Non-treated
PARGi
PARGi
Non-treated
U2OS
HEK293T
HeLa
Ln229
RD

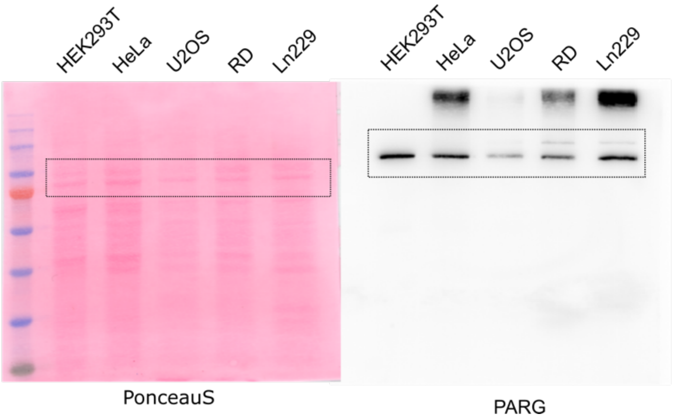
HEK293T
HeLa
U2OS
RD
Ln229
HEK293T
HeLa
U2OS
RD
Ln229
PonceauS
PARG
